# The effect of healthy eating on the development of stomach and colorectal cancer by the smoking and drinking status: Results from the Korean National Cancer Center (KNCC) community cohort study

**DOI:** 10.1002/cam4.70053

**Published:** 2024-08-22

**Authors:** Yuri Han, Jin‐Kyoung Oh, Min Kyung Lim

**Affiliations:** ^1^ Department of Social and Preventive Medicine, College of Medicine Inha University Incheon Republic of Korea; ^2^ Department of Cancer Control and Population Health National Cancer Center Graduate School of Cancer Science and Policy Goyang Republic of Korea

**Keywords:** cohort, colorectal cancer, drinking, effect of food intake, smoking, stomach cancer

## Abstract

**Background:**

Determining the effect of dietary factors on cancer is a crucial issue when accounting for the effect of other major risks, such as smoking and drinking.

**Method:**

A total of 15,563 adults from the Korean National Cancer Center Community Cohort were analyzed to determine and to compare the effect of dietary factors on stomach and colorectal cancer in overall and in the subgroup of non‐smokers (or urinary cotinine concentrations <5 ng/mg) and non‐drinkers with Cox proportional‐hazard models.

**Results:**

During the mean follow‐up (13.7 years), 469 and 299 cases of stomach and colorectal cancer were identified, respectively. The preventive effect of vegetable, fish, and soybean/tofu intake on colorectal cancer was found in women after adjustment for smoking, drinking, BMI, and sociodemographic factors. In the subgroup analysis of non‐smokers and non‐drinkers, the effect on colorectal cancer was increased in women (≥1 time/week vs. almost never, vegetables: hazard ratio (HR) 0.30, 95% confidence interval (CI) 0.13–0.69; fish: HR 0.46, 95% CI 0.26–0.83), and the fresh fish intake effect on stomach cancer was newly identified in men (HR 0.36, 95% CI 0.15–0.86). These effects were more pronounced and additionally shown in other dietary factors such as soybean or tofu in women and vegetables and fish in men, when subjects with <5 ng/mg urinary cotinine concentrations applied.

**Conclusion:**

The protective effect of healthy eating on the risk of stomach and colorectal cancer were different by smoking and drinking status. Rigorous control of smoking and drinking effects is necessary when measuring the effect of dietary factors on cancer, properly.

## 1 INTRODUCTION

Stomach and colorectal cancer are the most common cancers worldwide; there were more than 1 million new cases of stomach cancer and more than 1.9 million new cases of colorectal cancer in 2020.^1^ In Korea, the incidence of stomach and colorectal cancer has been consistently high for the past 20 years: out of 10 cancers in 2020, stomach and colorectal cancer had the second and fourth highest incidence among men and the fifth and third highest incidence among women, respectively.^2^

Many studies have examined the associations among lifestyle factors, such as diet, smoking and drinking, and the prevention, intervention, and risk of stomach and colorectal cancers. The World Cancer Research Fund International has noted the harmful effects of red meat, processed meat, and foods preserved by salting and the beneficial effects of dietary fiber‐rich foods.^3^ Previous studies have also reported that gastric and colorectal cancers show negative associations with soy‐based foods, including soybean, tofu, miso, and soymilk, which are prominent in Asian (especially Korean and Japanese) cuisines;^4^, ^5^ fruit; and vegetables^6^, ^7^ and positive associations with pickled foods,^8^, ^9^ fermented soy foods,^4^ and red and processed meat.^10^, ^11^ Moreover, fish intake was found to have no association with stomach cancer risk^12^ but a negative association with colorectal cancer risk.^13^

Several studies have reported differences in the intake of foods and nutrients by smoking and drinking status. Smokers have shown lower intake of fiber, folate, vitamin C, and carotene than never smokers,^14^, ^15^ and alcohol intake of more than 10 g/day has been associated with lower intake of fruit, vegetables, dairy products (only among women), and dietary fiber and higher intake of processed meat and salt.^16^ Even though most previous nutritional epidemiological studies have calculated risk estimates of dietary factors adjusted for smoking and drinking, it is not enough to exclude the interaction or confounding effect of smoking and drinking on dietary factors as those two exposures are substantially correlated with dietary factors even with inconsistent direction. However, to the best of our knowledge, only several cohort studies have evaluated the effect of dietary factors on cancers by smoking status,^17^, ^18^ and few have evaluated the effect of dietary factors by drinking status.

Therefore, based on the community‐based prospective cohort study, the current study tried to measure the effect of dietary factors on the development of stomach and colorectal cancer, in which the variety of preventive or deleterious effect of dietary factors has been suggested, with the subgroup analysis by the smoking and drinking status as well as statistical adjustment of smoking and drinking in the multiple logistic regression model. As well, urinary cotinine concentration was also applied as an objective measure of smoking exposure together with topography of tobacco use based on the self‐report with the structured questionnaire.

## 2 METHODS

### 2.1 Study population

This study used baseline data obtained from the Korean National Cancer Center (KNCC) Community Cohort study, which recruited adults over 30 years of age living in the community (Sancheong, Uiryeong, Haman, Changwon, Chuncheon, Chungju) between 1993 and 2010. All participants provided written informed consent prior to participating in the study. Baseline data on sociodemographic factors and lifestyle factors, including frequency of food intake, smoking status, and drinking status, were collected by trained interviewers using a questionnaire. Blood and urine samples and anthropometric information were also collected. The design of the KNCC Community Cohort study has been previously published.^19^

The KNCC Community Cohort study included 16,304 participants at baseline. Of these, 15,563 participants were finally included in the analysis, excluding participants with cancer before enrollment (*n* = 297) and those under 30 years old (*n* = 444).

### 2.2 Assessment of food intake and other variables

The self‐administered food frequency questionnaire examined intake of 28 items, including vegetables, fruits, meat, fish, red meat, and soybean/tofu. Participants were asked to share their average intake frequency for each food item using seven categories (almost never, 1 time/month, 2–3 times/month, 1 time/week, 2–3 times/week, 4–6 times/week, 1 time/day, ≥2 times/day). We grouped these categories into almost never, ≤2–3 times/month, and ≥1 time/week. Based on the results from previous studies, more frequent consumption of vegetables, fruits, soybean or tofu, and fishes, and less frequent consumption of red meats are considered as “healthy eating” in this study.

Age was classified as <50, 50–59, 60–69, or ≥70. Education level was classified as illiterate, middle school, or high school or more. Marital status was classified as single or married. Body mass index (BMI) was determined by dividing body weight by the square of height (kg/m^2^) and then used to categorize participants as normal weight (<25 kg/m^2^) or obese (≥25 kg/m^2^) according to the International Obesity Task Force for adults in the Asia‐Pacific region.^20^ Drinking and smoking status were classified as never, former, or current. Urinary cotinine levels were measured by liquid chromatography–tandem mass spectrometry (using modified methods that have been previously described)^21^ and classified as <5 or ≥5 ng/mg.^22^ The limit of detection for cotinine was 2 ng/mg. Creatinine levels in the urine were measured via colorimetry (Toshiba 2090 FR; Toshiba, Tokyo, Japan) and used to calculate the creatinine‐adjusted cotinine concentration.

### 2.3 Definition of cancer cases

Cancer cases were identified by linking the cohort data with the Korea Central Cancer Registry data up to the end of 2018 using the personal identification numbers of participants who agreed to the linkage to secondary data, such as the cancer registry. Stomach (C16) and colorectal (C18, C19, C20) cancer were defined using the International Classification of Diseases, 10th revision.

### 2.4 Statistical analysis

We calculated the mean and standard deviation (SD) for continuous variables. For categorical variables, we calculated prevalence or percentages and used the Chi‐square test to analyze the differences among groups. We performed Cox proportional hazard regression analysis to estimate adjusted hazard ratios (HRs), 95% confidence intervals (CIs), and *p* for trend for risk of stomach and colorectal cancer according to food intake by sex. For each participant, we calculated person‐years of follow‐up from baseline to diagnosis of cancer, death, or end of follow‐up (31 December 2018), whichever occurred first. We used a crude model and two adjusted models: model 1 was adjusted for age, education, marital status, BMI, drinking status, and smoking status, and model 2 was adjusted for age, education, marital status, BMI, drinking status, and urinary cotinine levels. Subgroup analysis was conducted for the never‐drinking and never‐smoking groups to control for drinking and smoking, which are major risk factors for stomach and colorectal cancer. Because smoking exposure assessed using biomarkers is clearer than that assessed using the questionnaire, we used two subgroups: (1) self‐reported never and former smokers and never and former drinkers, and (2) participants with urinary cotinine levels of <5 ng/mg and self‐reported never and former drinkers. Additionally, a subgroup analysis was performed for smokers or drinkers: (1) self‐reported current smokers or current drinkers, and (2) participants with urinary cotinine levels of ≥5 ng/mg or self‐reported current drinkers.

*p*‐values less than 0.05 were considered statistically significant. All analyses were performed using SAS version 9.4 (SAS Institute Inc., Cary, NC, USA).

## 3 RESULTS

A total of 15,563 participants over 30 years of age (men: 6021, women: 9542) were included in the analysis. During a mean follow‐up period of 13.7 years, there were 469 stomach cancer cases (men: 296, women: 173) and 299 colorectal cancer cases (men: 148, women: 151). Table 1 presents the baseline characteristics of the study participants stratified by sex. The average age of the participants was approximately 59 years. The education levels were middle school or higher for most men (middle school: 60.8%, high school or more: 27.4%) and middle school or lower for most women (illiterate: 32.3%, middle school: 56.4%). The majority of men had a BMI of <25 kg/m^2^ (73.2%) and urinary cotinine of ≥5 ng/mg (57.8%) and were current drinkers (62.2%) and current smokers (48.4%). Most women also had a BMI of <25 kg/m^2^ (63.0%) but were never drinkers (80.2%) and never smokers (90.9%) and had a urinary cotinine level of <5 ng/mg (77.6%). Most participants consumed all foods ≥1 time/week, except red meat; approximately 91% of men consumed red meat more than 2–3 times/month, whereas approximately 71% of women consumed it 2–3 times/month or less.

**TABLE 1:** Baseline characteristics of study participants.

	Men (*n* = 6021)	Women (*n* = 9542)	*p*‐value
Cases of stomach cancer	296 (4.9)	173 (1.8)	
Cases of colorectal cancer	148 (2.5)	151 (1.6)	
Age (years), mean ± SD	59.2 ± 10.9	58.8 ± 11.2	
<50	1215 (20.2)	2107 (22.1)	0.0748
50–59	1617 (26.9)	2415 (25.3)	
60–69	2106 (35.0)	3372 (35.3)	
≥70	1083 (18.0)	1648 (17.3)	
Education level			
Illiterate	676 (11.9)	2947 (32.3)	<0.0001
Middle school	3466 (60.8)	5147 (56.4)	
High school or more	1560 (27.4)	1025 (11.2)	
Marital status			
Single	403 (6.8)	2997 (32.0)	<0.0001
Married	5506 (93.2)	6373 (68.0)	
BMI, mean ± SD	23.2 ± 2.9	24.1 ± 3.3	
<25 kg/m^2^, *n* (%)	4345 (73.2)	5940 (63.0)	<0.0001
≥25 kg/m^2^, *n* (%)	1590 (26.8)	3492 (37.0)	
Drinking status			
Never	1555 (26.2)	7478 (80.2)	<0.0001
Former	692 (11.6)	252 (2.7)	
Current	3699 (62.2)	1598 (17.1)	
Smoking status			
Never	1192 (20.0)	8568 (90.9)	<0.0001
Former	1884 (31.6)	237 (2.5)	
Current	2886 (48.4)	617 (6.6)	
Urinary cotinine (ng/mg)			
<5	1971 (42.2)	5834 (77.6)	<0.0001
≥5	2702 (57.8)	1685 (22.4)	
Vegetables (frequency)			
Almost never	79 (1.7)	155 (2.1)	<0.0001
≤2–3 times/month	251 (5.4)	563 (7.4)	
≥1 time/week	4305 (92.9)	6861 (90.5)	
Fruit (frequency)			
Almost never	298 (6.4)	485 (6.4)	0.0510
≤2–3 times/month	931 (19.8)	1664 (21.9)	
≥1 time/week	3466 (73.8)	5448 (71.7)	
Fish (frequency)			
Almost never	237 (5.2)	510 (6.9)	<0.0001
≤2–3 times/month	1422 (31.1)	2743 (37.2)	
≥1 time/week	2913 (63.7)	4125 (55.9)	
Red meat (frequency)			
Almost never	304 (9.2)	1207 (21.7)	<0.0001
≤2–3 times/month	1279 (38.8)	2722 (49.0)	
≥1 time/week	1715 (52.0)	1623 (29.2)	
Soybean/tofu (frequency)			
Almost never	133 (3.2)	308 (4.8)	0.0001
≤2–3 times/month	1152 (28.1)	1885 (29.3)	
≥1 time/week	2821 (68.7)	4244 (65.9)	

Table 2 presents the HRs (95% CIs) for the associations between food intake frequency and risk of stomach and colorectal cancer after adjustment for age, education level, marital status, BMI, drinking status, and self‐reported smoking status (or urinary cotinine level in model 2). Among men, both model 1 and model 2 showed a significant negative dose–response association between soybean/tofu intake and the risk of stomach cancer (model 1: *p* for trend 0.0014; model 2: *p* for trend 0.0144). Compared with almost never, a significant association was observed between salted fish intake and increased risk of colorectal cancer in model 1 (≤2–3 times/month: HR 2.04, 95% CI 1.13–3.67; ≥1 time/week: HR 1.80, 95% CI 1.01–3.21), but this association was only present for 2–3 times/month in model 2 (HR 1.92, 95% CI 1.05–3.51). Among women, in both models, ≥1 time/week intake of vegetables, fish, and red meat was significantly associated with a reduced risk of colorectal cancer (vegetables: HR 0.32, 95% CI 0.14–0.75; fish: HR 0.46, 95% CI 0.25–0.82; red meat: HR 0.49, 95% CI 0.24–1.00). ≥1 time/week intake of soybean/tofu was associated with a reduced risk of colorectal cancer in model 2 (HR 0.45, 95% CI 0.21–0.96), only. No significant associations were observed for stomach cancer.

**TABLE 2:** Hazard ratios (HRs) and 95% confidence intervals (95% CIs) for stomach and colorectal cancer by frequency of food intake.

	Total	Stomach cancer							Colorectal cancer						
		Cancer (%)	HR (95% CI)^a^	*p* for trend	HR (95% CI)^b^	*p* for trend	HR (95% CI)^c^	*p* for trend	Cancer (%)	HR (95% CI)^a^	*p* for trend	HR (95% CI)^b^	*p* for trend	HR (95% CI)^c^	*p* for trend
*Men*															
Vegetables															
Almost never	79 (1.7)	4 (5.1)	Ref.	0.6595	Ref.	0.6021	Ref.	0.4505	2 (2.5)	Ref.	0.5850	Ref.	0.7799	Ref.	0.8014
≤2–3 times/month	251 (5.4)	9 (3.6)	0.71 (0.22–2.29)		0.80 (0.25–2.60)		0.83 (0.22–3.13)		7 (2.8)	1.08 (0.23–5.21)		1.13 (0.23–5.45)		0.85 (0.17–4.25)	
≥1 time/week	4305 (92.9)	214 (5.0)	0.94 (0.35–2.52)		1.01 (0.37–2.72)		1.14 (0.36–3.59)		97 (2.3)	0.86 (0.21–3.48)		0.98 (0.24–4.00)		0.82 (0.20–3.38)	
Fruit															
Almost never	298 (6.4)	12 (4.0)	Ref.	0.1090	Ref.	0.6418	Ref.	0.6428	6 (2.0)	Ref.	0.3792	Ref.	0.8211	Ref.	0.8479
≤2–3 times/month	931 (19.8)	61 (6.6)	1.51 (0.81–2.80)		1.54 (0.82–2.86)		1.57 (0.76–3.24)		27 (2.9)	1.34 (0.55–3.24)		1.41 (0.58–3.43)		1.40 (0.53–3.73)	
≥1 time/week	3466 (73.8)	153 (4.4)	1.00 (0.56–1.80)		1.21 (0.67–2.18)		1.20 (0.60–2.38)		76 (2.2)	0.98 (0.43–2.24)		1.16 (0.50–2.68)		1.16 (0.46–2.92)	
Fish															
Almost never	237 (5.2)	11 (4.6)	Ref.	0.1884	Ref.	0.4813	Ref.	0.2292	3 (1.3)	Ref.	0.7907	Ref.	0.9672	Ref.	0.7100
≤2–3 times/month	1422 (31.1)	85 (6.0)	1.28 (0.69–2.41)		1.27 (0.68–2.39)		1.15 (0.59–2.25)		42 (3.0)	2.36 (0.73–7.62)		2.24 (0.69–7.28)		2.06 (0.63–6.74)	
≥1 time/week	2913 (63.7)	131 (4.5)	0.99 (0.53–1.82)		1.05 (0.56–1.97)		0.88 (0.46–1.71)		67 (2.3)	1.82 (0.57–5.80)		1.88 (0.58–6.03)		1.59 (0.49–5.16)	
Fresh fish															
Almost never	525 (13.9)	36 (6.9)	Ref.	0.0205	Ref.	0.1091	Ref.	0.1090	16 (3.1)	Ref.	0.2855	Ref.	0.5084	Ref.	0.7630
≤2–3 times/month	1214 (32.1)	73 (6.0)	0.91 (0.61–1.35)		0.94 (0.62–1.40)		1.07 (0.61–1.88)		36 (3.0)	1.02 (0.56–1.84)		0.98 (0.54–1.78)		1.38 (0.60–3.16)	
≥1 time/week	2043 (54.0)	87 (4.3)	0.67 (0.45–0.99)		0.75 (0.50–1.12)		0.75 (0.43–1.30)		47 (2.3)	0.79 (0.45–1.40)		0.85 (0.47–1.52)		1.11 (0.49–2.51)	
Salted fish															
Almost never	1102 (22.9)	49 (4.5)	Ref.	0.4971	Ref.	0.7503	Ref.	0.7664	15 (1.4)	Ref.	0.1966	Ref.	0.1187	Ref.	0.1832
≤2–3 times/month	1578 (32.8)	93 (5.9)	1.20 (0.85–1.69)		1.19 (0.84–1.70)		1.26 (0.86–1.86)		46 (2.9)	1.99 (1.11–3.57)		2.04 (1.13–3.67)		1.92 (1.05–3.51)	
≥1 time/week	2136 (44.4)	95 (4.5)	0.94 (0.67–1.33)		0.98 (0.69–1.39)		0.99 (0.67–1.46)		51 (2.4)	1.68 (0.94–2.98)		1.80 (1.01–3.21)		1.66 (0.92–3.00)	
Red meat															
Almost never	304 (9.2)	8 (2.6)	Ref.	0.8923	Ref.	0.6852	Ref.	0.8248	10 (3.3)	Ref.	0.4149	Ref.	0.9528	Ref.	0.9724
≤2–3 times/month	1279 (38.8)	50 (3.9)	1.38 (0.65–2.90)		1.53 (0.72–3.23)		1.48 (0.70–3.14)		28 (2.2)	0.60 (0.29–1.23)		0.65 (0.31–1.35)		0.62 (0.29–1.29)	
≥1 time/week	1715 (52.0)	59 (3.4)	1.19 (0.57–2.48)		1.40 (0.66–2.97)		1.31 (0.61–2.78)		41 (2.4)	0.63 (0.32–1.26)		0.79 (0.39–1.60)		0.77 (0.38–1.58)	
Beef															
Almost never	1355 (41.2)	53 (3.9)	Ref.	0.3859	Ref.	0.4032	Ref.	0.6301	33 (2.4)	Ref.	0.6906	Ref.	0.9997	Ref.	0.9492
≤2–3 times/month	1326 (40.3)	44 (3.3)	0.84 (0.56–1.25)		0.85 (0.57–1.28)		0.87 (0.57–1.32)		33 (2.5)	1.00 (0.62–1.62)		1.10 (0.67–1.80)		0.99 (0.60–1.65)	
≥1 time/week	611 (18.6)	20 (3.3)	0.83 (0.50–1.39)		0.84 (0.49–1.43)		0.92 (0.54–1.58)		13 (2.1)	0.86 (0.45–1.63)		0.96 (0.50–1.85)		0.98 (0.51–1.89)	
Pork															
Almost never	392 (11.9)	12 (3.1)	Ref.	0.9329	Ref.	0.5912	Ref.	0.7445	10 (2.6)	Ref.	0.7263	Ref.	0.5902	Ref.	0.6073
≤2–3 times/month	1254 (38.0)	47 (3.8)	1.16 (0.61–2.18)		1.31 (0.69–2.47)		1.26 (0.66–2.40)		29 (2.3)	0.84 (0.41–1.72)		0.95 (0.46–1.96)		0.89 (0.43–1.85)	
≥1 time/week	1651 (50.1)	58 (3.5)	1.06 (0.57–1.97)		1.28 (0.68–2.42)		1.19 (0.62–2.26)		40 (2.4)	0.84 (0.42–1.69)		1.11 (0.54–2.26)		1.08 (0.53–2.20)	
Soybean/tofu															
Almost never	133 (3.2)	7 (5.3)	Ref.	0.0016	Ref.	0.0014	Ref.	0.0144	3 (2.3)	Ref.	0.1722	Ref.	0.3272	Ref.	0.4670
≤2–3 times/month	1152 (28.1)	87 (7.6)	1.14 (0.52–2.46)		1.22 (0.56–2.65)		1.36 (0.49–3.80)		21 (1.8)	0.70 (0.21–2.36)		0.76 (0.22–2.58)		0.78 (0.18–3.49)	
≥1 time/week	2821 (68.7)	100 (3.5)	0.67 (0.31–1.45)		0.70 (0.32–1.51)		0.80 (0.29–2.20)		68 (2.4)	1.08 (0.34–3.42)		1.02 (0.32–3.29)		1.05 (0.25–4.34)	
*Women*															
Vegetables															
Almost never	155 (2.1)	2 (1.3)	Ref.	0.4166	Ref.		Ref.		7 (4.5)	Ref.	0.0006	Ref.	0.0007	Ref.	0.0009
≤2–3 times/month	563 (7.4)	7 (1.2)	0.91 (0.19–4.37)		‐		‐		13 (2.3)	0.48 (0.19–1.21)		0.53 (0.21–1.32)		0.63 (0.24–1.68)	
≥1 time/week	6861 (90.5)	123 (1.8)	1.27 (0.31–5.12)		‐		‐		98 (1.4)	0.29 (0.13–0.62)		0.30 (0.14–0.65)		0.32 (0.14–0.75)	
Fruit															
Almost never	485 (6.4)	9 (1.9)	Ref.	0.2058		0.8395	Ref.	0.9795	9 (1.9)	Ref.	0.0806	Ref.	0.2998	Ref.	0.4598
≤2–3 times/month	1664 (21.9)	35 (2.1)	1.08 (0.52–2.26)		1.14 (0.54–2.39)		0.89 (0.40–2.00)		32 (1.9)	1.00 (0.48–2.10)		1.14 (0.52–2.48)		1.04 (0.44–2.42)	
≥1 time/week	5448 (71.7)	86 (1.6)	0.81 (0.41–1.62)		1.12 (0.56–2.26)		0.93 (0.44–1.96)		73 (1.3)	0.70 (0.35–1.39)		0.86 (0.41–1.82)		0.85 (0.38–1.90)	
Fish															
Almost never	510 (6.9)	7 (1.4)	Ref.	0.5565		0.4623	Ref.	0.0755	18 (3.5)	Ref.	0.0125	Ref.	0.0621	Ref.	0.0522
≤2–3 times/month	2743 (37.2)	57 (2.1)	1.41 (0.64–3.10)		1.76 (0.75–4.10)		1.53 (0.60–3.91)		43 (1.6)	0.42 (0.24–0.73)		0.49 (0.28–0.86)		0.45 (0.25–0.83)	
≥1 time/week	4125 (55.9)	64 (1.6)	1.13 (0.52–2.46)		1.69 (0.72–3.93)		1.99 (0.79–5.02)		58 (1.4)	0.40 (0.24–0.68)		0.48 (0.28–0.84)		0.46 (0.25–0.82)	
Fresh fish															
Almost never	1137 (18.6)	22 (1.9)	Ref.	0.7936		0.2153	Ref.	0.0553	29 (2.6)	Ref.	0.1181	Ref.	0.2231	Ref.	0.0989
≤2–3 times/month	2153 (35.3)	39 (1.8)	0.98 (0.58–1.65)		1.03 (0.60–1.76)		0.94 (0.43–2.02)		36 (1.7)	0.69 (0.42–1.13)		0.73 (0.44–1.20)		0.58 (0.32–1.05)	
≥1 time/week	2816 (46.1)	49 (1.7)	1.05 (0.63–1.75)		1.35 (0.80–2.29)		1.57 (0.76–3.24)		41 (1.5)	0.66 (0.41–1.07)		0.71 (0.43–1.17)		0.56 (0.31–1.01)	
Salted fish															
Almost never	1891 (24.1)	29 (1.5)	Ref.	0.3275		0.9966	Ref.	0.6550	35 (1.9)	Ref.	0.1264	Ref.	0.2428	Ref.	0.1649
≤2–3 times/month	2804 (35.8)	62 (2.2)	1.18 (0.76–1.85)		1.25 (0.79–1.97)		1.01 (0.62–1.66)		43 (1.5)	0.69 (0.44–1.09)		0.75 (0.48–1.19)		0.73 (0.45–1.19)	
≥1 time/week	3139 (40.1)	45 (1.4)	0.84 (0.53–1.35)		1.03 (0.64–1.66)		1.11 (0.67–1.82)		44 (1.4)	0.69 (0.44–1.07)		0.75 (0.47–1.18)		0.70 (0.42–1.15)	
Red meat															
Almost never	1207 (21.7)	15 (1.2)	Ref.	0.6056		0.1748	Ref.	0.2922	28 (2.3)	Ref.	0.0003	Ref.	0.0290	Ref.	0.0336
≤2–3 times/month	2722 (49.0)	47 (1.7)	1.28 (0.72–2.30)		1.81 (0.99–3.34)		1.67 (0.91–3.08)		36 (1.3)	0.53 (0.32–0.86)		0.66 (0.39–1.11)		0.62 (0.36–1.04)	
≥1 time/week	1623 (29.2)	20 (1.2)	0.89 (0.46–1.74)		1.70 (0.84–3.46)		1.52 (0.74–3.12)		13 (0.8)	0.31 (0.16–0.60)		0.47 (0.24–0.96)		0.49 (0.24–1.00)	
Beef															
Almost never	2744 (49.5)	41 (1.5)	Ref.	0.5667		0.5668	Ref.	0.5315	45 (1.6)	Ref.	0.1156	Ref.	0.4513	Ref.	0.3886
≤2–3 times/month	2180 (39.3)	34 (1.6)	1.01 (0.64–1.59)		1.25 (0.78–2.00)		1.27 (0.79–2.04)		25 (1.2)	0.67 (0.41–1.10)		0.79 (0.48–1.31)		0.71 (0.42–1.19)	
≥1 time/week	623 (11.2)	7 (1.1)	0.73 (0.33–1.62)		1.07 (0.47–2.42)		1.08 (0.48–2.47)		7 (1.1)	0.66 (0.30–1.46)		0.84 (0.37–1.89)		0.88 (0.39–2.00)	
Pork															
Almost never	1473 (26.6)	20 (1.4)	Ref.	0.4802		0.2541	Ref.	0.4269	31 (2.1)	Ref.	0.0009	Ref.	0.0656	Ref.	0.0926
≤2–3 times/month	2570 (46.3)	44 (1.7)	1.18 (0.69–1.99)		1.59 (0.91–2.75)		1.46 (0.84–2.54)		34 (1.3)	0.59 (0.36–0.95)		0.73 (0.44–1.22)		0.72 (0.43–1.21)	
≥1 time/week	1506 (27.1)	18 (1.2)	0.80 (0.42–1.51)		1.47 (0.75–2.87)		1.31 (0.67–2.59)		12 (0.8)	0.34 (0.18–0.67)		0.53 (0.26–1.08)		0.57 (0.28–1.16)	
Soybean/tofu															
Almost never	308 (4.8)	5 (1.6)	Ref.	0.2747		0.4860	Ref.	0.8950	9 (2.9)	Ref.	0.2440	Ref.	0.2689	Ref.	0.0973
≤2–3 times/month	1185 (29.3)	49 (2.6)	1.24 (0.49–3.13)		1.57 (0.62–3.96)		1.12 (0.39–3.25)		34 (1.8)	0.49 (0.23–1.02)		0.53 (0.25–1.12)		0.50 (0.22–1.14)	
≥1 time/week	4244 (65.9)	61 (1.4)	0.93 (0.38–2.32)		1.18 (0.47–2.96)		1.04 (0.38–2.90)		59 (1.4)	0.50 (0.25–1.00)		0.54 (0.26–1.09)		0.45 (0.21–0.96)	

^a^ Crude.

^b^ Adjusted for age, education, marital status, smoking status, drinking status, BMI.

^c^ Adjusted for age, education, marital status, urinary cotinine level, drinking status, BMI.

Table 3 shows the adjusted HRs (95% CI) for the associations between food intake and risk of stomach and colorectal cancer in the subgroup with self‐reported non‐smokers and non‐drinkers. This analysis produced different results compared with the overall analysis (Table 2). Among men, fruit and fresh fish intake had a negative dose–response association for stomach cancer (fruit: *p* for trend 0.0392; fresh fish: *p* for trend 0.0324), whereas salted fish intake had a positive dose–response association for colorectal cancer (*p* for trend 0.0433). Among women, compared with almost never, ≥1 time/week intake of red meat increased the risk of stomach cancer (HR 2.32, 95% CI 1.07–5.30). More frequent intake of vegetables and fish reduced the risk of colorectal cancer (vegetables: HR 0.30, 95% CI 0.13–0.69; fish: HR 0.46, 95% CI 0.26–0.83). However, the effect of soybean or tofu intake on stomach cancer in men and vegetables intake on stomach cancer in women could not be estimated because no cancer cases were incident in the category of “almost never.”

**TABLE 3:** Hazard ratios (HRs) and 95% confidence intervals (95% CIs) for stomach and colorectal cancer by frequency of food intake among self‐reported non‐smokers and non‐drinkers.

	Total	Stomach cancer					Colorectal cancer				
		Cancer (%)	HR (95% CI)^a^	*p* for trend	HR (95% CI)^b^	*p* for trend	Cancer (%)	HR (95% CI)^a^	*p* for trend	HR (95% CI)^b^	*p* for trend
*Men*											
Vegetables											
Almost never	16 (1.6)	2 (12.5)	Ref.	0.2618		0.2837	1 (6.3)	Ref.	0.1311	Ref.	0.2086
≤2–3 times/month	58 (5.7)	2 (3.5)	0.25 (0.04–1.76)		0.28 (0.04–2.01)		2 (3.5)	0.39 (0.04–4.36)		0.31 (0.03–3.56)	
≥1 time/week	940 (92.7)	38 (4.0)	0.29 (0.07–1.21)		0.29 (0.07–1.22)		15 (1.6)	0.23 (0.03–1.70)		0.23 (0.03–1.86)	
Fruit											
Almost never	39 (3.8)	2 (5.1)	Ref.	0.0276		0.0392	1 (2.6)	Ref.	0.1418	Ref.	0.1865
≤2–3 times/month	202 (19.4)	14 (6.9)	1.02 (0.23–4.50)		0.97 (0.22–4.35)		6 (3.0)	0.96 (0.12–7.98)		1.02 (0.12–8.61)	
≥1 time/week	798 (76.8)	27 (3.4)	0.49 (0.12–2.06)		0.46 (0.11–1.97)		13 (1.6)	0.47 (0.06–3.61)		0.53 (0.07–4.09)	
Fish											
Almost never	55 (5.6)	4 (7.3)	Ref.	0.5710		0.4984	1 (1.8)	Ref.	0.9653	Ref.	0.9728
≤2–3 times/month	338 (34.5)	13 (3.9)	0.53 (0.17–1.62)		0.43 (0.14–1.35)		7 (2.1)	1.25 (0.15–10.19)		1.41 (0.17–11.51)	
≥1 time/week	586 (59.9)	23 (3.9)	0.57 (0.20–1.64)		0.47 (0.16–1.40)		12 (2.1)	1.19 (0.16–9.18)		1.29 (0.17–10.07)	
Fresh fish											
Almost never	117 (14.8)	10 (8.6)	Ref.	0.0458		0.0324	5 (4.3)	Ref.	0.0513	Ref.	0.0530
≤2–3 times/month	278 (35.2)	10 (3.6)	0.46 (0.19–1.11)		0.44 (0.18–1.07)		7 (2.5)	0.63 (0.20–2.00)		0.68 (0.21–2.16)	
≥1 time/week	396 (50.1)	11 (2.8)	0.39 (0.16–0.93)		0.36 (0.15–0.86)		5 (1.3)	0.30 (0.09–1.04)		0.31 (0.09–1.07)	
Salted fish											
Almost never	266 (25.3)	11 (4.1)	Ref.	0.9391		0.8911	1 (0.4)	Ref.	0.0358	Ref.	0.0433
≤2–3 times/month	372 (35.3)	16 (4.3)	0.94 (0.44–2.05)		0.88 (0.40–1.91)		8 (2.2)	5.85 (0.73–46.78)		6.50 (0.81–52.12)	
≥1 time/week	415 (39.4)	17 (4.1)	0.96 (0.45–2.07)		0.93 (0.43–2.00)		11 (2.7)	7.65 (0.99–59.21)		7.63 (0.98–59.38)	
Red meat											
Almost never	95 (12.7)	2 (2.1)	Ref.	0.8854		0.9443	2 (2.1)	Ref.	0.3282	Ref.	0.4753
≤2–3 times/month	342 (45.5)	13 (3.8)	1.67 (0.38–7.41)		1.76 (0.40–7.83)		7 (2.1)	0.86 (0.18–4.15)		0.83 (0.17–4.14)	
≥1 time/week	314 (41.8)	9 (2.9)	1.23 (0.27–5.69)		1.37 (0.29–6.44)		4 (1.3)	0.49 (0.09–2.70)		0.59 (0.11–3.34)	
Beef											
Almost never	292 (38.9)	10 (3.4)	Ref.	0.6037		0.6713	5 (1.7)	Ref.	0.3630	Ref.	0.3458
≤2–3 times/month	333 (44.3)	11 (3.3)	0.92 (0.39–2.18)		0.97 (0.41–2.30)		8 (2.4)	1.30 (0.42–3.99)		1.32 (0.43–4.11)	
≥1 time/week	126 (16.8)	3 (2.4)	0.70 (0.19–2.53)		0.75 (0.20–2.77)		0	–		–	
Pork											
Almost never	129 (17.2)	3 (2.3)	Ref.	0.9431		0.7628	2 (1.6)	Ref.	0.5842	Ref.	0.8430
≤2–3 times/month	327 (43.5)	12 (3.7)	1.50 (0.42–5.32)		1.55 (0.44–5.54)		7 (2.1)	1.27 (0.26–6.14)		1.49 (0.30–7.27)	
≥1 time/week	295 (39.3)	9 (3.1)	1.22 (0.33–4.49)		1.36 (0.36–5.14)		4 (1.4)	0.74 (0.14–4.08)		1.03 (0.18–5.77)	
Soybean/tofu											
Almost never	31 (3.4)	0	Ref.		Ref.		1 (3.3)	Ref.	0.4146	Ref.	0.6835
≤2–3 times/month	254 (27.7)	20 (7.9)	‐		‐		3 (1.2)	0.34 (0.04–3.30)		0.34 (0.03–3.29)	
≥1 time/week	632 (68.9)	20 (3.2)	‐		‐		13 (2.1)	0.66 (0.09–5.01)		0.58 (0.08–4.54)	
*Women*											
Vegetables											
Almost never	102 (1.8)	0	Ref.		Ref.		6 (5.9)	Ref.	0.0002	Ref.	0.0017
≤2–3 times/month	398 (7.0)	7 (1.8)	‐		‐		11 (2.8)	0.44 (0.16–1.20)		0.52 (0.19–1.42)	
≥1 time/week	5220 (91.3)	91 (1.7)	‐		‐		84 (1.6)	0.24 (0.10–0.54)		0.30 (0.13–0.69)	
Fruit											
Almost never	319 (5.6)	5 (1.6)	Ref.	0.4314	Ref.	0.9661	7 (2.2)	Ref.	0.0383	Ref.	0.2191
≤2–3 times/month	1181 (20.6)	25 (2.1)	1.25 (0.48–3.27)		1.37 (0.52–3.60)		28 (2.4)	1.02 (0.45–2.34)		1.11 (0.48–2.56)	
≥1 time/week	4238 (73.9)	68 (1.6)	0.96 (0.39–2.37)		1.17 (0.47–2.94)		62 (1.5)	0.63 (0.29–1.38)		0.79 (0.36–1.74)	
Fish											
Almost never	390 (6.9)	3 (0.8)	Ref.	0.4776	Ref.	0.1404	17 (4.4)	Ref.	0.0088	Ref.	0.0717
≤2–3 times/month	2079 (37.0)	42 (2.0)	2.45 (0.76–7.91)		2.60 (0.80–8.44)		37 (1.8)	0.39 (0.22–0.69)		0.45 (0.25–0.82)	
≥1 time/week	3147 (56.0)	55 (1.8)	2.29 (0.72–7.32)		2.79 (0.87–8.99)		50 (1.6)	0.37 (0.21–0.64)		0.46 (0.26–0.83)	
Fresh fish											
Almost never	849 (17.9)	15 (1.8)	Ref.	0.3486	Ref.	0.1315	28 (3.3)	Ref.	0.0667	Ref.	0.2169
≤2–3 times/month	1676 (35.3)	30 (1.8)	1.06 (0.57–1.98)		1.10 (0.59–2.05)		30 (1.8)	0.58 (0.34–0.97)		0.63 (0.37–1.06)	
≥1 time/week	2223 (46.8)	42 (1.9)	1.29 (0.71–2.33)		1.51 (0.83–2.76)		37 (1.7)	0.59 (0.36–0.98)		0.69 (0.41–1.16)	
Salted fish											
Almost never	1460 (24.6)	16 (1.1)	Ref.	0.6545	Ref.	0.3335	30 (2.1)	Ref.	0.1721	Ref.	0.3652
≤2–3 times/month	2133 (36.0)	49 (2.3)	1.67 (0.95–2.96)		1.71 (0.96–3.03)		38 (1.8)	0.72 (0.44–1.16)		0.78 (0.48–1.27)	
≥1 time/week	2338 (39.4)	37 (1.6)	1.29 (0.72–2.32)		1.44 (0.80–2.61)		37 (1.6)	0.70 (0.43–1.13)		0.78 (0.47–1.28)	
Red meat											
Almost never	925 (22.6)	8 (0.9)	Ref.	0.9574	Ref.	0.2620	23 (2.5)	Ref.	0.0046	Ref.	0.0778
≤2–3 times/month	2023 (49.4)	36 (1.8)	1.90 (0.88–4.08)		2.32 (1.07–5.03)		27 (1.3)	0.50 (0.29–0.87)		0.60 (0.34–1.07)	
≥1 time/week	1150 (28.1)	13 (1.1)	1.17 (0.48–2.81)		1.80 (0.73–4.43)		12 (1.0)	0.38 (0.19–0.76)		0.54 (0.26–1.13)	
Beef											
Almost never	2031 (49.6)	26 (1.3)	Ref.	0.7939	Ref.	0.4211	34 (1.7)	Ref.	0.3725	Ref.	0.6948
≤2–3 times/month	1606 (39.2)	25 (1.6)	1.18 (0.68–2.04)		1.31 (0.75–2.27)		22 (1.4)	0.79 (0.46–1.36)		0.87 (0.51–1.50)	
≥1 time/week	458 (11.2)	6 (1.3)	0.99 (0.41–2.41)		1.26 (0.51–3.10)		6 (1.3)	0.76 (0.32–1.81)		0.92 (0.38–2.23)	
Pork											
Almost never	1132 (27.6)	12 (1.1)	Ref.	0.7723	Ref.	0.4254	26 (2.3)	Ref.	0.0078	Ref.	0.1215
≤2–3 times/month	1909 (46.6)	34 (1.8)	1.56 (0.81–3.01)		1.89 (0.97–3.67)		25 (1.3)	0.53 (0.31–0.92)		0.65 (0.37–1.13)	
≥1 time/week	1055 (25.8)	11 (1.0)	0.88 (0.39–2.00)		1.36 (0.59–3.14)		11 (1.0)	0.41 (0.20–0.83)		0.59 (0.28–1.25)	
Soybean/tofu											
Almost never	222 (4.5)	3 (1.4)	Ref.	0.4241	Ref.	0.4675	7 (3.2)	Ref.	0.4676	Ref.	0.5592
≤2–3 times/month	1498 (30.6)	40 (2.7)	1.45 (0.45–4.71)		1.74 (0.53–5.65)		29 (1.9)	0.46 (0.20–1.05)		0.52 (0.22–1.20)	
≥1 time/week	3172 (64.8)	45 (1.4)	1.10 (0.34–3.53)		1.26 (0.39–4.09)		49 (1.5)	0.51 (0.23–1.12)		0.57 (0.26–1.28)	

^a^ Crude.

^b^ Adjusted for age, education, marital status, BMI.

Table 4 shows the adjusted HRs (95%CI) for the associations between food intake and risk of cancer in the subgroup of participants with urinary cotinine levels of <5 ng/mg and self‐reported never drinkers. Overall, especially in men, the associations were slightly more evident than those in the first subgroup (Table 3), and more foods were significantly associated with cancer risk. Among men, ≥1 time/week intake of vegetables, fish, and fresh fish was strongly associated with stomach cancer risk (vegetables: HR 0.18, 95% CI 0.04–0.81; fish: HR 0.27, 95% CI 0.08–0.87; fresh fish: HR 0.17, 95% CI 0.04–0.69). There was a negative dose–response association between fruit intake and stomach cancer (*p* for trend 0.0104). Among women, ≤2–3 times/month intake of red meat increased the risk of stomach cancer, whereas ≥1 time/week intake of vegetables, fish, fresh fish, and soybean/tofu was significantly associated with reduced risk of colorectal cancer (vegetables: HR 0.32, 95% CI 0.11–0.88; fish: HR 0.44, 95% CI 0.22–0.87; fresh fish: HR 0.53, 95% CI 0.27–1.04; soybean/tofu: HR 0.41, 95% CI 0.17–0.98).

**TABLE 4:** Hazard ratios (HRs) and 95% confidence intervals (95% CIs) for stomach and colorectal cancer by frequency of food intake among urinary cotinine level <5 ng/mg and non‐drinkers.

	Total	Stomach cancer					Colorectal cancer				
		Cancer (%)	HR (95% CI)^a^	*p* for trend	HR (95% CI)^b^	*p* for trend	Cancer (%)	HR (95% CI)^a^	*p* for trend	HR (95% CI)^b^	*p* for trend
*Men*											
Vegetables											
Almost never	12 (1.7)	2 (16.7)	Ref.	0.0465	Ref.	0.0748	1 (8.3)	Ref.	0.0872	Ref.	0.1215
≤2–3 times/month	40 (5.8)	1 (2.5)	0.12 (0.01–1.35)		0.18 (0.02–2.07)		1 (2.5)	0.19 (0.01–3.11)		0.15 (0.01–2.56)	
≥1 time/week	644 (92.5)	18 (2.8)	0.14 (0.03–0.60)		0.18 (0.04–0.81)		10 (1.6)	0.13 (0.02–0.99)		0.13 (0.02–1.08)	
Fruit											
Almost never	25 (3.5)	2 (8.0)	Ref.	0.0034	Ref.	0.0104	0	Ref.		Ref.	
≤2–3 times/month	133 (18.8)	8 (6.0)	0.66 (0.14–3.13)		0.62 (0.13–3.01)		5 (3.8)	‐		‐	
≥1 time/week	548 (77.6)	11 (2.0)	0.21 (0.05–0.94)		0.22 (0.05–1.05)		9 (1.6)	‐		‐	
Fish											
Almost never	42 (6.5)	4 (9.5)	Ref.	0.1293	Ref.	0.1144	1 (2.4)	Ref.	0.5313	Ref.	0.5378
≤2–3 times/month	216 (33.2)	5 (2.3)	0.26 (0.07–0.98)		0.26 (0.07–0.99)		6 (2.8)	1.31 (0.16–10.87)		1.22 (0.15–10.17)	
≥1 time/week	393 (60.4)	10 (2.5)	0.29 (0.09–0.91)		0.27 (0.08–0.87)		7 (1.8)	0.82 (0.10–6.68)		0.85 (0.10–7.04)	
Fresh fish											
Almost never	56 (11.5)	4 (7.1)	Ref.	0.0208	Ref.	0.0175	3 (5.4)	Ref.	0.0306	Ref.	0.0380
≤2–3 times/month	152 (31.2)	5 (3.3)	0.45 (0.12–1.68)		0.43 (0.11–1.65)		5 (3.3)	0.60 (0.14–2.50)		0.60 (0.14–2.55)	
≥1 time/week	280 (57.4)	4 (1.4)	0.20 (0.05–0.78)		0.17 (0.04–0.69)		3 (1.1)	0.19 (0.04–0.95)		0.21 (0.04–1.10)	
Salted fish											
Almost never	208 (29.1)	9 (4.3)	Ref.	0.5189	Ref.	0.5118	1 (0.5)	Ref.	0.1738	Ref.	0.2014
≤2–3 times/month	233 (32.6)	5 (2.2)	0.52 (0.17–1.54)		0.52 (0.17–1.56)		7 (3.0)	6.53 (0.80–53.07)		6.55 (0.80–53.47)	
≥1 time/week	273 (38.2)	8 (2.9)	0.73 (0.28–1.89)		0.70 (0.27–1.83)		6 (2.2)	5.10 (0.61–42.34)		5.00 (0.60–41.69)	
Red meat											
Almost never	80 (13.8)	1 (1.3)	Ref.	0.8623	Ref.	0.6233	1 (1.3)	Ref.	0.9676	Ref.	0.7371
≤2–3 times/month	263 (45.2)	9 (3.4)	2.60 (0.33–20.55)		2.90 (0.37–22.94)		5 (1.9)	1.48 (0.17–12.65)		1.37 (0.16–12.17)	
≥1 time/week	239 (41.1)	6 (2.5)	1.90 (0.23–15.81)		2.41 (0.29–20.31)		4 (1.7)	1.25 (0.14–11.15)		1.56 (0.17–14.37)	
Beef											
Almost never	232 (39.9)	7 (3.0)	Ref.	0.9605	Ref.	0.8401	4 (1.7)	Ref.	0.4897	Ref.	0.4873
≤2–3 times/month	260 (44.7)	6 (2.3)	0.74 (0.25–2.21)		0.84 (0.28–2.54)		6 (2.3)	1.32 (0.37–4.69)		1.31 (0.36–4.70)	
≥1 time/week	90 (15.5)	3 (3.3)	1.11 (0.29–4.29)		1.31 (0.33–5.18)		0	‐		‐	
Pork											
Almost never	109 (18.7)	2 (1.8)	Ref.	0.8085	Ref.	0.5704	1 (0.9)	Ref.	0.7110	Ref.	0.4855
≤2–3 times/month	247 (42.4)	8 (3.2)	1.72 (0.37–8.09)		1.93 (0.41–9.11)		5 (2.0)	2.18 (0.26–18.69)		2.33 (0.27–20.15)	
≥1 time/week	226 (38.8)	6 (2.7)	1.41 (0.29–7.00)		1.83 (0.36–9.22)		4 (1.8)	1.84 (0.21–16.50)		2.51 (0.27–23.03)	
Soybean/tofu											
Almost never	21 (3.3)	0	Ref.		Ref.		1 (4.8)	Ref.	0.4146	Ref.	0.4097
≤2–3 times/month	134 (21.2)	7 (5.2)	‐		‐		3 (2.2)	0.47 (0.05–4.51)		0.41 (0.04–4.16)	
≥1 time/week	476 (75.4)	11 (2.3)	‐		‐		8 (1.7)	0.37 (0.05–2.99)		0.31 (0.04–2.57)	
*Women*											
Vegetables											
Almost never	68 (1.6)	0	Ref.		Ref.		4 (5.9)	Ref.	0.0006	Ref.	0.0029
≤2–3 times/month	304 (7.2)	5 (1.6)	‐		‐		10 (3.3)	0.54 (0.17–1.73)		0.66 (0.21–2.11)	
≥1 time/week	3835 (91.2)	66 (1.7)	‐		‐		57 (1.5)	0.24 (0.09–0.67)		0.32 (0.11–0.88)	
Fruit											
Almost never	219 (5.2)	4 (1.8)	Ref.	0.5583	Ref.	0.9131	5 (2.3)	Ref.	0.0523	Ref.	0.2635
≤2–3 times/month	818 (19.4)	15 (1.8)	0.91 (0.30–2.76)		1.00 (0.33–3.01)		18 (2.2)	0.93 (0.35–2.50)		1.04 (0.38–2.80)	
≥1 time/week	3170 (75.4)	51 (1.6)	0.80 (0.29–2.22)		1.01 (0.36–2.83)		43 (1.4)	0.56 (0.22–1.42)		0.74 (0.29–1.90)	
Fish											
Almost never	268 (6.6)	2 (0.8)	Ref.	0.2773	Ref.	0.0504	13 (4.9)	Ref.	0.0023	Ref.	0.0516
≤2–3 times/month	1445 (35.7)	27 (1.9)	2.30 (0.55–9.69)		2.56 (0.60–10.87)		27 (1.9)	0.38 (0.19–0.73)		0.49 (0.25–0.97)	
≥1 time/week	2331 (57.6)	43 (1.8)	2.51 (0.61–10.35)		3.41 (0.82–14.24)		33 (1.4)	0.30 (0.16–0.57)		0.44 (0.22–0.87)	
Fresh fish											
Almost never	390 (12.0)	6 (1.5)	Ref.	0.3215	Ref.	0.0870	15 (3.9)	Ref.	0.0087	Ref.	0.1023
≤2–3 times/month	1164 (35.9)	19 (1.6)	0.86 (0.34–2.18)		0.89 (0.35–2.28)		23 (2.0)	0.46 (0.24–0.89)		0.57 (0.29–1.12)	
≥1 time/week	1693 (52.1)	35 (2.1)	1.24 (0.52–2.94)		1.54 (0.64–3.73)		26 (1.5)	0.38 (0.20–0.72)		0.53 (0.27–1.04)	
Salted fish											
Almost never	1169 (26.9)	14 (1.2)	Ref.	0.6468	Ref.	0.3567	25 (2.1)	Ref.	0.0637	Ref.	0.1953
≤2–3 times/month	1492 (34.4)	34 (2.3)	1.52 (0.81–2.85)		1.48 (0.78–2.81)		28 (1.9)	0.77 (0.45–1.33)		0.85 (0.49–1.47)	
≥1 time/week	1680 (38.7)	26 (1.6)	1.24 (0.65–2.38)		1.39 (0.72–2.68)		21 (1.3)	0.58 (0.32–1.03)		0.68 (0.38–1.23)	
Red meat											
Almost never	803 (23.3)	7 (0.9)	Ref.	0.8755	Ref.	0.3564	22 (2.7)	Ref.	0.0069	Ref.	0.1119
≤2–3 times/month	1709 (49.6)	32 (1.9)	2.02 (0.89–4.58)		2.47 (1.08–5.64)		21 (1.2)	0.42 (0.23–0.77)		0.52 (0.28–0.95)	
≥1 time/week	933 (27.1)	9 (1.0)	1.02 (0.38–2.74)		1.63 (0.59–4.49)		11 (1.2)	0.40 (0.19–0.82)		0.59 (0.27–1.28)	
Beef											
Almost never	1735 (50.4)	22 (1.3)	Ref.	0.8591	Ref.	0.4137	32 (1.8)	Ref.	0.2251	Ref.	0.5091
≤2–3 times/month	1335 (38.8)	22 (1.7)	1.28 (0.71–2.31)		1.44 (0.79–2.61)		17 (1.3)	0.67 (0.37–1.21)		0.74 (0.41–1.35)	
≥1 time/week	373 (10.8)	4 (1.1)	0.84 (0.29–2.44)		1.16 (0.39–3.43)		5 (1.3)	0.71 (0.28–1.82)		0.92 (0.35–2.41)	
Pork											
Almost never	983 (28.6)	11 (1.1)	Ref.	0.6546	Ref.	0.5494	24 (2.4)	Ref.	0.0146	Ref.	0.1881
≤2–3 times/month	1605 (46.6)	29 (1.8)	1.52 (0.76–3.04)		1.82 (0.90–3.68)		20 (1.3)	0.48 (0.27–0.87)		0.59 (0.32–1.08)	
≥1 time/week	855 (24.8)	8 (0.9)	0.77 (0.31–1.92)		1.20 (0.47–3.07)		10 (1.2)	0.44 (0.21–0.92)		0.67 (0.31–1.45)	
Soybean/tofu											
Almost never	151 (4.3)	3 (2.0)	Ref.	0.5678	Ref.	0.7223	6 (4.0)	Ref.	0.1537	Ref.	0.2025
≤2–3 times/month	870 (24.5)	21 (2.4)	0.77 (0.22–2.64)		0.90 (0.26–3.11)		14 (1.6)	0.32 (0.12–0.86)		0.38 (0.14–1.02)	
≥1 time/week	2529 (71.2)	37 (1.5)	0.70 (0.22–2.28)		0.83 (0.26–2.72)		36 (1.4)	0.35 (0.15–0.83)		0.41 (0.17–0.98)	

^a^ Crude.

^b^ Adjusted for age, education, marital status, BMI.

Tables S1 and S2 show the adjusted HRs (95%CI) for the associations between food intake and risk of cancer in the subgroup of participants with self‐reported current smokers (or urinary cotinine levels of ≥5 ng/mg) or current drinkers. Among men, soybean/tofu intake had a negative dose–response association for stomach cancer (subgroup of participants with self‐reported current smokers or current drinkers: *p* for trend 0.0021, subgroup of participants with urinary cotinine levels of ≥5 ng/mg or current drinkers: *p* for trend 0.0040), which is similar to the analysis results in overall subjects. Among women, negative association between vegetable intake and colorectal cancer incidence was shown (≥1 time/week vs. almost never HR 0.20, 95%CI 0.04–0.99), which is similar with the results from overall subjects and subgroup with non‐smokers and non‐drinkers.

## 4 DISCUSSION

Our study identified differences in the effects of dietary factors on the risk of stomach and colorectal cancer between the results from the models adjusted for smoking and drinking as confounding factors and the results from subgroups of non‐smokers and non‐drinkers that restricted these factors. We found that in assessments of smoking exposure, associations evaluated based on urinary cotinine levels were stronger than those evaluated based on self‐reported smoking status. In the model adjusted for urinary cotinine levels and other factors, salted fish (men only), soybean/tofu (men and women), vegetable (women only), fish (women only), and red meat (women only) intake were significantly associated with a reduced risk of cancer. However, in the subgroup with self‐reported non‐smokers and non‐drinkers, fruit (men only), fresh fish (men only), vegetable (women only), and fish (women only) intake were associated with a reduced risk of cancer, and the significant association was more pronounced in the subgroup of never drinkers with urinary cotinine levels of <5 ng/mg. This subgroup also showed a significant association between red meat (women only) and soybean/tofu (women only) intake and reduced risk of cancer.

Several previous studies have evaluated the effects of dietary factors on stomach and colorectal cancer risk in subgroups restricted to self‐reported smoking status, and the effects differed from those adjusted for self‐reported smoking status. But few studies have evaluated the effects in subgroups that restricted drinking factors as well. The Shanghai Women's and Men's Health Studies found that intake of all fruits was not associated with distal gastric cancer risk (Q4 vs. Q1 HR 0.79, 95% CI 0.48–1.30, *p* for trend 0.23), but intake of all fruits except watermelon was associated with a reduced risk of distal gastric cancer in men (Q4 vs. Q1 HR 0.50, 95% CI 0.29–0.84, *p* for trend 0.004). The significant negative association was maintained in ever smokers (Q4 vs. Q1 HR 0.43, 95% CI 0.26–0.79, *p* for trend 0.001) but not in never smokers (Q4 vs. Q1 HR 0.77, 95% CI 0.27–2.23, *p* for trend 0.67).^17^ In a case–control study, despite considering *Helicobacter pylori* infection, which was not considered in our study, fruit and vegetable intake was not associated with stomach cancer risk in men (fruit: 108.83 vs. 78.55 g/day HR 1.14, 95% CI 0.53–2.46, *p* for trend 0.736; vegetables: 89.99 vs. 62.97 g/day HR 0.50, 95% CI 0.22–1.12, *p* for trend 0.094), but never smokers with high fruit and vegetable intake showed a reduced risk of stomach cancer compared with ever smokers with low fruit and vegetable intake (fruit: HR 0.20, 95% CI 0.06–0.65; vegetables: HR 0.25, 95% CI 0.06–0.95).^23^ Similarly, our study did not find fruit and vegetable intake to be associated with stomach cancer risk in the overall cohort, but it was associated with a reduced risk of stomach cancer among men in all subgroups.

In the European Prospective Investigation into Cancer and Nutrition study, vegetable intake was not associated with colorectal cancer risk (Q5 vs. Q1 HR 0.92, 95% CI 0.79–1.06, *p* for trend 0.27), except for a negative association among former smokers (Q5 vs. Q1 HR 0.69, 95% CI 0.53–0.89, *p* for trend <0.01) and a positive association among current smokers (Q5 vs. Q1 HR 1.41, 95% CI 1.03–1.93, *p* for trend <0.01).^18^ Our study also found a significant association between vegetable intake and reduced risk of colorectal cancer, including in all subgroups, but only among women.

A cohort study in Korea suggested that soybean/tofu intake was associated with a reduced risk of stomach cancer in women (men: high intake vs. low intake HR 0.77, 95% CI 0.52–1.13; women: high intake vs. low intake HR 0.41, 95% CI 0.22–0.78), but there was no statistically significant association among male smokers and never smokers (*p* for interaction 0.268).^24^ Our study showed a significant dose–response association between soybean/tofu intake and reduced risk of stomach cancer among men, but not in all subgroups. Another cohort study in China and Japan found that dietary isoflavone and soy protein intake were not significantly associated with colorectal cancer risk, but when stratified by smoking status, dietary soy protein intake was significantly associated with a reduced risk of colorectal cancer in ever smokers (Q4 vs. Q1 HR 0.81, 95% CI 0.67–0.99).^5^ By contrast, our study found a significant association between soybean/tofu intake and reduced risk of colorectal cancer in the model adjusted for urinary cotinine levels and other factors and among women in the subgroup of never drinkers with urinary cotinine of <5 ng/mg.

Previous studies have evaluated the association between fish intake and the risk of stomach and colorectal cancer, but few have reported the association after restricting smoking and drinking factors. A meta‐analysis study suggested that fish intake was not associated with stomach cancer risk.^12^ Our study also found no such association; however, among men, fresh fish intake was associated with a reduced risk of stomach cancer in all subgroups. Other systematic review and meta‐analysis studies have found a significant association between fish intake and reduced risk of colorectal cancer.^13^, ^25^ Our study found that this association was significant in women, including in all subgroups.

As such, the beneficial effects of vegetable, fruit, fish, fresh fish, and soybean/tofu intake on stomach and colorectal cancer risk are more evident in subgroups, particularly in men, which can be explained as follows. First, previous studies have suggested that never smokers and never drinkers generally have healthier diets than smokers and drinkers: never smokers and never drinkers have shown higher intake of oily fish, tofu, tofu products, n‐3 and n‐6 polyunsaturated fatty acids, fiber, carotene, folate, total antioxidant status, and vitamins C and E than smokers and drinkers, who tend to show higher intake of alcohol, carbohydrates, and sodium as well as a higher total oxidant status and oxidative stress index than never smokers and never drinkers.^14^, ^15^, ^16^ In our study, non‐smokers and non‐drinkers had higher fruit, vegetable (only women), and fresh fish (only women) intake than current smokers and current drinkers (data not shown). Second, a cross‐sectional study evaluated the synergistic interaction of smoking and drinking in the reduction of serum carotenoids (lycopene, a‐carotene, b‐carotene, lutein, b‐cryptoxanthin, and zeaxanthin) and found that the average serum carotenoid concentrations did not differ between never smokers and current smokers after adjusting for age and sex in never drinkers. However, daily alcohol intake reduced the serum carotenoid concentrations (a‐carotene, b‐carotene, and b‐cryptoxanthin) in a dose‐dependent manner, and these concentrations were obviously lower in current smokers than in never smokers.^26^ Furthermore, another study found that passive smokers had a significantly lower plasma antioxidant status than never smokers, regardless of differences in dietary antioxidant intake.^27^

Previous studies have applied self‐reported smoking status to control for smoking factors, but to our knowledge, no study has controlled for smoking exposure using biomarkers. Studies comparing self‐reported smoking status and cotinine levels have reported a significantly lower prevalence of self‐reported smoking than that assessed by urinary cotinine levels.^28^, ^29^ Moreover, the 2009–2018 Korean National Health and Nutrition Examination Survey showed a discrepancy between self‐reported secondhand smoke exposure and that measured by urinary cotinine among never smokers aged 19 or older.^30^ Therefore, to address the possibility of misclassification by self‐reported smoking status, we performed a subgroup analysis to examine both self‐reported non‐smokers and individuals with urinary cotinine levels less than 5 ng/mg. The preventive effects of fruit, vegetable, fish, and fresh fish intake in the subgroup of never drinkers with urinary cotinine levels less than 5 ng/mg were clearly associated with a reduced risk of cancer compared with the subgroup of self‐reported non‐smokers and non‐drinkers in men, but the significant associations in the two subgroups were similar in women. These results are unsurprising for several reasons. First, the self‐reported non‐smokers may include hidden smokers, particularly in women. Second, we included former smokers as self‐reported never smokers due to small sample sizes, and former smokers who quit abruptly for health or other reasons may have been included. Third, urinary cotinine levels of <5 ng/mg represent a lack of exposure to either secondhand smoke or active smoke, thereby controlling exposure to smoking more completely than the self‐reports. In our study, self‐reported non‐smokers, those with urinary cotinine concentrations of <5 ng/mg, and never drinkers accounted for 51.6%, 42.7%, and 37.8% of men and 82.9%, 77.6%, and 93.4% of women, respectively; thus, differences between the results of the overall cohort and the subgroups may have been more pronounced in men than in women.

Although red meat intake is a well‐established risk factor for colorectal cancer,^3^ the literature has called for more epidemiologic studies considering Korean dietary culture.^31^ Our results found a significant association between red meat intake and reduced risk of colorectal cancer in women. Our participants were recruited from the community, mostly from rural areas where meat intake is relatively low. Of the women who participated, 21.7% and 49.0% reported red meat intake of almost never and ≤2–3 times/month, respectively. Thus, we expect that the association is due to the low frequency of red meat intake and the small sample size.

Our study has several limitations. First, food intake data were collected at baseline; changes in dietary habits during the follow‐up period were not considered. Second, our data did not include information on food intake in grams per day, menopausal status, and *Helicobacter pylori* infection, a major risk factor for stomach cancer. Third, we identified 469 stomach cancer cases and 299 colorectal cancer cases during a mean follow‐up period of 13.7 years, and the sample sizes of the subgroups of these cases were rather small. As the reason, further analysis on the effect of dietary factors by the smoking and drinking group categorized by amount and duration exposed could not be done, and the effect of some dietary factors were not calculated in women with current smokers or current drinkers (Table S1 and S2). Fourth, our subjects were 58 years old on average, lived in the community (mostly in rural areas), and had a low frequency of red meat intake (<1 time/week; men: 48.0%, women: 70.7%), so the association of red meat intake with cancer risk could not be effectively evaluated. Despite these limitations, this prospective cohort study identified the different effects of food intake on stomach and colorectal cancer risk by smoking and drinking status, particularly by applying both self‐reported smoking status and smoking biomarkers, and confirmed the difference in between two groups.

## 5 CONCLUSION

The current study investigated the effect of vegetable, fruit, fish, and soybean/tofu intake on the risk of stomach and colorectal cancer. The protective effect of healthy eating on the risk of those cancer were identified but different by smoking and drinking status. The effect was more pronounced in the subgroup of non‐drinkers with <5 ng/mg urinary cotinine concentrations than among non‐drinkers and self‐reported non‐smokers.

Rigorous control of smoking and drinking effects looks necessary when measuring the effect of dietary factors on cancer, properly, although further study is in need to confirm the current study results with the lager sample followed up longer period.

## AUTHOR CONTRIBUTIONS

**Yuri Han:** Formal analysis (lead); investigation (equal); writing – original draft (equal); writing – review and editing (equal). **Jin‐Kyoung Oh:** Investigation (equal); writing – review and editing (equal). **Min Kyung Lim:** Conceptualization (lead); supervision (lead); writing – original draft (equal); writing – review and editing (equal).

## FUNDING INFORMATION

This study was financially supported by Inha University (68648–1,71341‐1) and NRF‐2020R1A2C2012295.

## CONFLICT OF INTEREST STATEMENT

The authors declare no conflicts of interest.

## ETHICS STATEMENT

All cohort participants provided written informed consent before the baseline survey. The study protocols were approved by the Institutional Review Board of the Seoul National University Hospital (IRB number: 740‐C508) and the National Cancer Center of Korea (IRB number: NCCNHS02‐007; NCCNHS03‐081‐1; NCCNCS‐07‐080).

## Supporting information


Data S1.

## Data Availability

Data sharing is not applicable to this article as no new data were created or analyzed in this study.
