# Supplementary material for: The effect of healthy eating on the development of stomach and colorectal cancer by the smoking and drinking status: Results from the Korean National Cancer Center (KNCC) community cohort study
Source: Cancer Med. 2024 Aug 22;13(16):e70053. doi: 10.1002/cam4.70053 (PMC11339466; doi:10.1002/cam4.70053)
Supplement: Supplementary file 1 — Data S1. [file CAM4-13-e70053-s001.docx]

| **Supplementary Table 1. Hazard ratios (HRs) and 95% confidence intervals (95% CIs) for stomach and colorectal cancer by frequency of food intake among self-reported current smokers and current drinkers** | | | | | | | | | | | | |
| --- | --- | --- | --- | --- | --- | --- | --- | --- | --- | --- | --- | --- |
|  | **Total** | **Stomach cancer** | | | | |  | **Colorectal cancer** | | | | |
|  |  | **Cancer (%)** | **HR (95% CI)^1)^** | **p for trend** | **HR (95% CI)^2)^** | **p for trend** |  | **Cancer (%)** | **HR (95% CI)^1)^** | **p for trend** | **HR (95% CI)^2)^** | **p for trend** |
| **Men** |  |  |  |  |  |  |  |  |  |  |  |  |
| **Vegetables** |  |  |  |  |  |  |  |  |  |  |  |  |
| Almost never | 90 (1.8) | 2 (2.2) | Ref. | 0.0885 | Ref. | 0.0863 |  | 1 (1.1) | Ref. | 0.8529 | Ref. | 0.8289 |
| ≤2–3 times/month | 299 (6.0) | 7 (2.3) | 1.06 (0.22-5.11) |  | 1.19 (0.25-5.75) |  |  | 7 (2.3) | 2.12 (0.26-17.24) |  | 2.35 (0.29-19.19) |  |
| ≥1 time/week | 4,609 (92.2) | 198 (4.3) | 1.93 (0.48-7.77) |  | 2.06 (0.51-8.33) |  |  | 93 (2.0) | 1.82 (0.25-13.04) |  | 1.93 (0.27-13.92) |  |
| **Fruit** |  |  |  |  |  |  |  |  |  |  |  |  |
| Almost never | 362 (7.2) | 13 (3.6) | Ref. | 0.5040 | Ref. | 0.8523 |  | 5 (1.4) | Ref. | 0.9304 | Ref. | 0.6348 |
| ≤2–3 times/month | 1,063 (21.1) | 51 (4.8) | 1.30 (0.71-2.40) |  | 1.24 (0.67-2.29) |  |  | 25 (2.4) | 1.67 (0.64-4.37) |  | 1.61 (0.62-4.21) |  |
| ≥1 time/week | 3,604 (71.7) | 139 (3.9) | 1.04 (0.59-1.84) |  | 1.17 (0.66-2.07) |  |  | 72 (2.0) | 1.39 (0.56-3.45) |  | 1.51 (0.61-3.76) |  |
| **Fish** |  |  |  |  |  |  |  |  |  |  |  |  |
| Almost never | 247 (5.1) | 8 (3.2) | Ref. | 0.2241 | Ref. | 0.4621 |  | 3 (1.2) | Ref. | 0.5195 | Ref. | 0.8243 |
| ≤2–3 times/month | 1,539 (31.5) | 81 (5.3) | 1.60 (0.77-3.30) |  | 1.57 (0.76-3.25) |  |  | 41 (2.7) | 2.16 (0.67-6.98) |  | 2.14 (0.66-6.94) |  |
| ≥1 time/week | 3,098 (63.4) | 116 (3.7) | 1.17 (0.57-2.40) |  | 1.22 (0.59-2.51) |  |  | 59 (1.9) | 1.57 (0.49-5.00) |  | 1.71 (0.53-5.49) |  |
| **Fresh fish** |  |  |  |  |  |  |  |  |  |  |  |  |
| Almost never | 598 (15.2) | 28 (4.7) | Ref. | 0.3700 | Ref. | 0.7736 |  | 12 (2.0) | Ref. | 0.9302 | Ref. | 0.6841 |
| ≤2–3 times/month | 1,247 (31.6) | 69 (5.5) | 1.24 (0.80-1.92) |  | 1.26 (0.81-1.95) |  |  | 35 (2.8) | 1.47 (0.76-2.84) |  | 1.46 (0.76-2.83) |  |
| ≥1 time/week | 2,096 (53.2) | 82 (3.9) | 0.92 (0.60-1.43) |  | 1.02 (0.65-1.59) |  |  | 44 (2.1) | 1.14 (0.60-2.17) |  | 1.27 (0.66-2.46) |  |
| **Salted fish** |  |  |  |  |  |  |  |  |  |  |  |  |
| Almost never | 1,133 (21.9) | 46 (4.1) | Ref. | 0.1747 | Ref. | 0.4153 |  | 18 (1.6) | Ref. | 0.9496 | Ref. | 0.7326 |
| ≤2–3 times/month | 1,683 (32.6) | 84 (5.0) | 1.09 (0.76-1.56) |  | 1.11 (0.77-1.60) |  |  | 43 (2.6) | 1.45 (0.83-2.51) |  | 1.49 (0.85-2.59) |  |
| ≥1 time/week | 2,352 (45.5) | 85 (3.6) | 0.82 (0.57-1.18) |  | 0.89 (0.62-1.28) |  |  | 44 (1.9) | 1.10 (0.63-1.90) |  | 1.21 (0.70-2.11) |  |
| **Red meat** |  |  |  |  |  |  |  |  |  |  |  |  |
| Almost never | 380 (10.4) | 10 (2.6) | Ref. | 0.8114 | Ref. | 0.3254 |  | 11 (2.9) | Ref. | 0.4942 | Ref. | 0.7833 |
| ≤2–3 times/month | 1,481 (40.5) | 44 (3.0) | 1.06 (0.53-2.10) |  | 1.23 (0.61-2.46) |  |  | 28 (1.9) | 0.60 (0.30-1.21) |  | 0.71 (0.35-1.45) |  |
| ≥1 time/week | 1,793 (49.1) | 55 (3.1) | 1.08 (0.55-2.13) |  | 1.36 (0.68-2.74) |  |  | 38 (2.1) | 0.67 (0.34-1.30) |  | 0.93 (0.46-1.89) |  |
| **Beef** |  |  |  |  |  |  |  |  |  |  |  |  |
| Almost never | 1,566 (43.0) | 52 (3.3) | Ref. | 0.3522 | Ref. | 0.4271 |  | 35 (2.2) | Ref. | 0.8430 | Ref. | 0.7314 |
| ≤2–3 times/month | 1,464 (40.2) | 40 (2.7) | 0.81 (0.54-1.22) |  | 0.82 (0.54-1.24) |  |  | 28 (1.9) | 0.84 (0.51-1.38) |  | 0.95 (0.57-1.57) |  |
| ≥1 time/week | 616 (16.9) | 17 (2.8) | 0.82 (0.48-1.42) |  | 0.86 (0.49-1.51) |  |  | 14 (2.3) | 1.01 (0.54-1.87) |  | 1.16 (0.61-2.20) |  |
| **Pork** |  |  |  |  |  |  |  |  |  |  |  |  |
| Almost never | 475 (13.0) | 14 (3.0) | Ref. | 0.8610 | Ref. | 0.2967 |  | 11 (2.3) | Ref. | 0.7798 | Ref. | 0.4533 |
| ≤2–3 times/month | 1,447 (39.6) | 41 (2.8) | 0.91 (0.50-1.67) |  | 1.09 (0.59-2.01) |  |  | 29 (2.0) | 0.81 (0.40-1.62) |  | 0.97 (0.48-1.98) |  |
| ≥1 time/week | 1,731 (47.4) | 54 (3.1) | 1.00 (0.55-1.79) |  | 1.29 (0.70-2.39) |  |  | 37 (2.1) | 0.85 (0.43-1.67) |  | 1.22 (0.60-2.47) |  |
| **Soybean/tofu** |  |  |  |  |  |  |  |  |  |  |  |  |
| Almost never | 158 (3.6) | 8 (5.1) | Ref. | 0.0021 | Ref. | 0.0021 |  | 4 (2.5) | Ref. | 0.6845 | Ref. | 0.8090 |
| ≤2–3 times/month | 1,181 (26.9) | 73 (6.2) | 1.00 (0.48-2.07) |  | 0.94 (0.45-1.97) |  |  | 22 (1.9) | 0.66 (0.23-1.92) |  | 0.64 (0.22-1.89) |  |
| ≥1 time/week | 3,060 (69.6) | 91 (3.0) | 0.60 (0.29-1.24) |  | 0.56 (0.27-1.17) |  |  | 62 (2.0) | 0.82 (0.30-2.26) |  | 0.78 (0.28-2.16) |  |
| **Women** |  |  |  |  |  |  |  |  |  |  |  |  |
| **Vegetables** |  |  |  |  |  |  |  |  |  |  |  |  |
| Almost never | 15 (4.5) | 1 (6.7) | Ref. | 0.8491 | Ref. | 0.9944 |  | 1 (6.7) | Ref. |  | Ref. |  |
| ≤2–3 times/month | 28 (8.5) | 0 | - |  | - |  |  | 0 | - |  | - |  |
| ≥1 time/week | 288 (87.0) | 9 (3.1) | 0.46 (0.06-3.65) |  | 0.57 (0.07-4.55) |  |  | 1 (0.4) | - |  | - |  |
| **Fruit** |  |  |  |  |  |  |  |  |  |  |  |  |
| Almost never | 56 (16.8) | 1 (1.8) | Ref. | 0.9258 | Ref. | 0.9867 |  | 1 (1.8) | Ref. |  | Ref. |  |
| ≤2–3 times/month | 115 (34.4) | 5 (4.4) | 2.42 (0.28-20.69) |  | 2.62 (0.30-22.69) |  |  | 0 | - |  | - |  |
| ≥1 time/week | 163 (48.8) | 4 (2.5) | 1.37 (0.15-12.21) |  | 1.49 (0.16-13.57) |  |  | 1 (0.6) | - |  | - |  |
| **Fish** |  |  |  |  |  |  |  |  |  |  |  |  |
| Almost never | 42 (13.0) | 2 (4.8) | Ref. | 0.0901 | Ref. | 0.1125 |  | 0 | Ref. |  | Ref. |  |
| ≤2–3 times/month | 155 (47.8) | 5 (3.2) | 0.64 (0.12-3.29) |  | 0.58 (0.11-3.09) |  |  | 0 | - |  | - |  |
| ≥1 time/week | 127 (39.2) | 1 (0.8) | 0.15 (0.01-1.63) |  | 0.15 (0.01-1.80) |  |  | 2 (1.6) | - |  | - |  |
| **Fresh fish** |  |  |  |  |  |  |  |  |  |  |  |  |
| Almost never | 79 (28.8) | 4 (5.1) | Ref. | 0.1358 | Ref. | 0.1523 |  | 0 | Ref. |  | Ref. |  |
| ≤2–3 times/month | 118 (43.1) | 2 (1.7) | 0.32 (0.06-1.76) |  | 0.27 (0.05-1.58) |  |  | 0 | - |  | - |  |
| ≥1 time/week | 77 (28.1) | 1 (1.3) | 0.24 (0.03-2.13) |  | 0.26 (0.03-2.51) |  |  | 1 (1.3) | - |  | - |  |
| **Salted fish** |  |  |  |  |  |  |  |  |  |  |  |  |
| Almost never | 101 (29.2) | 4 (4.0) | Ref. | 0.1735 | Ref. | 0.2084 |  | 1 (1.0) | Ref. |  | Ref. |  |
| ≤2–3 times/month | 139 (40.2) | 5 (3.6) | 0.88 (0.24-3.26) |  | 0.81 (0.21-3.08) |  |  | 0 | - |  | - |  |
| ≥1 time/week | 106 (30.6) | 1 (0.9) | 0.22 (0.02-1.95) |  | 0.24 (0.03-2.20) |  |  | 1 (0.9) | - |  | - |  |
| **Red meat** |  |  |  |  |  |  |  |  |  |  |  |  |
| Almost never | 81 (40.7) | 2 (2.5) | Ref. | 0.8120 | Ref. | 0.7620 |  | 1 (1.2) | Ref. |  | Ref. |  |
| ≤2–3 times/month | 82 (41.2) | 4 (4.9) | 1.98 (0.36-10.82) |  | 2.90 (0.51-16.67) |  |  | 1 (1.2) | - |  | - |  |
| ≥1 time/week | 36 (18.1) | 1 (2.8) | 1.05 (0.10-11.52) |  | 1.05 (0.09-11.81) |  |  | 0 | - |  | - |  |
| **Beef** |  |  |  |  |  |  |  |  |  |  |  |  |
| Almost never | 142 (71.0) | 5 (3.5) | Ref. | 0.7382 | Ref. | 0.7261 |  | 2 (1.4) | Ref. |  | Ref. |  |
| ≤2–3 times/month | 44 (22.0) | 2 (4.6) | 1.35 (0.26-6.94) |  | 1.73 (0.31-9.59) |  |  | 0 | - |  | - |  |
| ≥1 time/week | 14 (7.0) | 0 | - |  | - |  |  | 0 | - |  | - |  |
| **Pork** |  |  |  |  |  |  |  |  |  |  |  |  |
| Almost never | 87 (43.9) | 2 (2.3) | Ref. | 0.6739 | Ref. | 0.5367 |  | 1 (1.2) | Ref. |  | Ref. |  |
| ≤2–3 times/month | 78 (39.4) | 4 (5.1) | 2.24 (0.41-12.25) |  | 2.99 (0.52-17.07) |  |  | 1 (1.3) | - |  | - |  |
| ≥1 time/week | 33 (16.7) | 1 (3.0) | 1.24 (0.11-13.70) |  | 1.49 (0.13-16.64) |  |  | 0 | - |  | - |  |
| **Soybean/tofu** |  |  |  |  |  |  |  |  |  |  |  |  |
| Almost never | 26 (9.6) | 1 (3.9) | Ref. | 0.6762 | Ref. | 0.5481 |  | 0 | Ref. |  | Ref. |  |
| ≤2–3 times/month | 91 (33.6) | 3 (3.3) | 0.92 (0.10-8.82) |  | 1.67 (0.17-16.76) |  |  | 1 (1.1) | - |  | - |  |
| ≥1 time/week | 154 (56.8) | 4 (2.6) | 0.70 (0.08-6.22) |  | 0.76 (0.08-6.99) |  |  | 1 (0.7) | - |  | - |  |
| 1) Crude | | | | | | | | | | | | |
| 2) Adjusted for age, education, marital status, BMI | | | | | | | | | | | | |

| **Supplementary Table 2. Hazard ratios (HRs) and 95% confidence intervals (95% CIs) for stomach and colorectal cancer by frequency of food intake among urinary cotinine level ≥5ng/mg and current drinkers** | | | | | | | | | | | | |
| --- | --- | --- | --- | --- | --- | --- | --- | --- | --- | --- | --- | --- |
|  | **Total** | **Stomach cancer** | | | | |  | **Colorectal cancer** | | | | |
|  |  | **Cancer (%)** | **HR (95% CI)^1)^** | **p for trend** | **HR (95% CI)^2)^** | **p for trend** |  | **Cancer (%)** | **HR (95% CI)^1)^** | **p for trend** | **HR (95% CI)^2)^** | **p for trend** |
| **Men** |  |  |  |  |  |  |  |  |  |  |  |  |
| **Vegetables** |  |  |  |  |  |  |  |  |  |  |  |  |
| Almost never | 91 (1.8) | 2 (2.2) | Ref. | 0.0626 | Ref. | 0.0602 |  | 1 (1.1) | Ref. | 0.9792 | Ref. | 0.9193 |
| ≤2–3 times/month | 312 (6.2) | 7 (2.2) | 1.02 (0.21-4.89) |  | 1.12 (0.23-5.38) |  |  | 8 (2.6) | 2.31 (0.29-18.47) |  | 2.47 (0.31-19.84) |  |
| ≥1 time/week | 4,651 (92.0) | 202 (4.3) | 1.98 (0.49-7.98) |  | 2.10 (0.52-8.48) |  |  | 93 (2.0) | 1.83 (0.26-13.14) |  | 1.94 (0.27-14.00) |  |
| **Fruit** |  |  |  |  |  |  |  |  |  |  |  |  |
| Almost never | 357 (7.0) | 12 (3.4) | Ref. | 0.7478 | Ref. | 0.6601 |  | 6 (1.7) | Ref. | 0.7504 | Ref. | 0.9620 |
| ≤2–3 times/month | 1,050 (20.6) | 50 (4.8) | 1.40 (0.74-2.63) |  | 1.33 (0.70-2.49) |  |  | 25 (2.4) | 1.41 (0.58-3.44) |  | 1.36 (0.56-3.32) |  |
| ≥1 time/week | 3,692 (72.4) | 146 (4.0) | 1.15 (0.64-2.07) |  | 1.27 (0.70-2.30) |  |  | 72 (2.0) | 1.13 (0.49-2.60) |  | 1.22 (0.53-2.82) |  |
| **Fish** |  |  |  |  |  |  |  |  |  |  |  |  |
| Almost never | 241 (4.9) | 8 (3.3) | Ref. | 0.1099 | Ref. | 0.2612 |  | 3 (1.2) | Ref. | 0.5118 | Ref. | 0.8235 |
| ≤2–3 times/month | 1,565 (31.6) | 85 (5.4) | 1.60 (0.78-3.31) |  | 1.58 (0.76-3.27) |  |  | 41 (2.6) | 2.07 (0.64-6.67) |  | 2.07 (0.64-6.70) |  |
| ≥1 time/week | 3,147 (63.5) | 116 (3.7) | 1.12 (0.55-2.28) |  | 1.16 (0.56-2.38) |  |  | 60 (1.9) | 1.52 (0.48-4.84) |  | 1.67 (0.52-5.36) |  |
| **Fresh fish** |  |  |  |  |  |  |  |  |  |  |  |  |
| Almost never | 558 (14.0) | 27 (4.8) | Ref. | 0.2034 | Ref. | 0.4108 |  | 11 (2.0) | Ref. | 1.000 | Ref. | 0.6883 |
| ≤2–3 times/month | 1,268 (31.8) | 71 (5.6) | 1.22 (0.78-1.90) |  | 1.20 (0.76-1.87) |  |  | 35 (2.8) | 1.49 (0.76-2.94) |  | 1.43 (0.72-2.83) |  |
| ≥1 time/week | 2,163 (54.2) | 82 (3.8) | 0.87 (0.56-1.34) |  | 0.91 (0.58-1.43) |  |  | 46 (2.1) | 1.18 (0.61-2.30) |  | 1.27 (0.65-2.51) |  |
| **Salted fish** |  |  |  |  |  |  |  |  |  |  |  |  |
| Almost never | 1,158 (22.1) | 46 (4.0) | Ref. | 0.1580 | Ref. | 0.3769 |  | 18 (1.6) | Ref. | 0.9095 | Ref. | 0.6023 |
| ≤2–3 times/month | 1,722 (32.8) | 90 (5.2) | 1.17 (0.82-1.67) |  | 1.20 (0.83-1.71) |  |  | 43 (2.5) | 1.45 (0.84-2.52) |  | 1.50 (0.86-2.61) |  |
| ≥1 time/week | 2,364 (45.1) | 84 (3.6) | 0.83 (0.58-1.19) |  | 0.90 (0.62-1.29) |  |  | 45 (1.9) | 1.14 (0.66-1.98) |  | 1.27 (0.73-2.21) |  |
| **Red meat** |  |  |  |  |  |  |  |  |  |  |  |  |
| Almost never | 392 (10.4) | 11 (2.8) | Ref. | 0.9697 | Ref. | 0.4679 |  | 12 (3.1) | Ref. | 0.2432 | Ref. | 0.8051 |
| ≤2–3 times/month | 1,545 (40.8) | 47 (3.0) | 1.01 (0.52-1.94) |  | 1.15 (0.59-2.24) |  |  | 30 (1.9) | 0.58 (0.30-1.13) |  | 0.69 (0.35-1.36) |  |
| ≥1 time/week | 1,848 (48.8) | 57 (3.1) | 1.01 (0.53-1.93) |  | 1.24 (0.63-2.41) |  |  | 37 (2.0) | 0.59 (0.31-1.12) |  | 0.81 (0.41-1.60) |  |
| **Beef** |  |  |  |  |  |  |  |  |  |  |  |  |
| Almost never | 1,611 (42.7) | 53 (3.3) | Ref. | 0.4402 | Ref. | 0.5100 |  | 36 (2.2) | Ref. | 0.7491 | Ref. | 0.8979 |
| ≤2–3 times/month | 1,523 (40.3) | 44 (2.9) | 0.86 (0.58-1.28) |  | 0.87 (0.58-1.31) |  |  | 29 (1.9) | 0.83 (0.51-1.36) |  | 0.93 (0.56-1.53) |  |
| ≥1 time/week | 643 (17.0) | 18 (2.8) | 0.84 (0.49-1.44) |  | 0.88 (0.51-1.51) |  |  | 14 (2.2) | 0.97 (0.52-1.79) |  | 1.08 (0.57-2.04) |  |
| **Pork** |  |  |  |  |  |  |  |  |  |  |  |  |
| Almost never | 492 (13.0) | 15 (3.1) | Ref. | 0.9712 | Ref. | 0.3934 |  | 12 (2.4) | Ref. | 0.4723 | Ref. | 0.7658 |
| ≤2–3 times/month | 1,512 (40.0) | 44 (2.9) | 0.90 (0.50-1.62) |  | 1.06 (0.59-1.92) |  |  | 31 (2.1) | 0.78 (0.40-1.52) |  | 0.95 (0.48-1.88) |  |
| ≥1 time/week | 1,780 (47.0) | 56 (3.2) | 0.96 (0.54-1.70) |  | 1.22 (0.67-2.21) |  |  | 36 (2.0) | 0.75 (0.39-1.45) |  | 1.07 (0.54-2.13) |  |
| **Soybean/tofu** |  |  |  |  |  |  |  |  |  |  |  |  |
| Almost never | 149 (3.4) | 7 (4.7) | Ref. | 0.0039 | Ref. | 0.0040 |  | 4 (2.7) | Ref. | 0.7099 | Ref. | 0.8634 |
| ≤2–3 times/month | 1,152 (26.0) | 73 (6.3) | 1.12 (0.51-2.43) |  | 1.07 (0.49-2.34) |  |  | 21 (1.8) | 0.63 (0.22-1.83) |  | 0.62 (0.21-1.84) |  |
| ≥1 time/week | 3,127 (70.6) | 96 (3.1) | 0.68 (0.31-1.46) |  | 0.65 (0.30-1.40) |  |  | 63 (2.0) | 0.79 (0.29-2.16) |  | 0.75 (0.27-2.07) |  |
| **Women** |  |  |  |  |  |  |  |  |  |  |  |  |
| **Vegetables** |  |  |  |  |  |  |  |  |  |  |  |  |
| Almost never | 41 (3.9) | 1 (2.4) | Ref. | 0.8301 | Ref. | 0.8418 |  | 2 (4.9) | Ref. | 0.0679 | Ref. | 0.1201 |
| ≤2–3 times/month | 110 (10.4) | 1 (0.9) | 0.34 (0.02-5.39) |  | 0.41 (0.03-6.65) |  |  | 1 (0.9) | 0.17 (0.02-1.92) |  | 0.24 (0.02-2.81) |  |
| ≥1 time/week | 909 (85.8) | 12 (1.3) | 0.53 (0.07-4.08) |  | 0.76 (0.10-6.01) |  |  | 8 (0.9) | 0.17 (0.04-0.82) |  | 0.20 (0.04-0.99) |  |
| **Fruit** |  |  |  |  |  |  |  |  |  |  |  |  |
| Almost never | 109 (10.1) | 2 (1.8) | Ref. | 0.0522 | Ref. | 0.1412 |  | 2 (1.8) | Ref. | 0.3299 | Ref. | 0.5085 |
| ≤2–3 times/month | 250 (23.1) | 7 (2.8) | 1.48 (0.31-7.12) |  | 1.30 (0.26-6.42) |  |  | 3 (1.2) | 0.62 (0.10-3.73) |  | 0.50 (0.08-3.15) |  |
| ≥1 time/week | 722 (66.8) | 6 (0.8) | 0.39 (0.08-1.95) |  | 0.46 (0.09-2.41) |  |  | 7 (1.0) | 0.46 (0.10-2.20) |  | 0.50 (0.10-2.54) |  |
| **Fish** |  |  |  |  |  |  |  |  |  |  |  |  |
| Almost never | 115 (10.4) | 2 (1.7) | Ref. | 0.7416 | Ref. | 0.5948 |  | 3 (2.6) | Ref. | 0.6886 | Ref. | 0.9307 |
| ≤2–3 times/month | 439 (39.6) | 3 (0.7) | 0.38 (0.06-2.30) |  | 0.36 (0.06-2.19) |  |  | 2 (0.5) | 0.17 (0.03-0.99) |  | 0.17 (0.03-1.04) |  |
| ≥1 time/week | 555 (50.1) | 8 (1.4) | 0.81 (0.17-3.81) |  | 0.92 (0.18-4.56) |  |  | 7 (1.3) | 0.45 (0.12-1.74) |  | 0.58 (0.14-2.36) |  |
| **Fresh fish** |  |  |  |  |  |  |  |  |  |  |  |  |
| Almost never | 157 (15.6) | 2 (1.3) | Ref. | 0.7894 | Ref. | 0.6576 |  | 3 (1.9) | Ref. | 0.8416 | Ref. | 0.9865 |
| ≤2–3 times/month | 389 (38.7) | 3 (0.8) | 0.59 (0.10-3.50) |  | 0.52 (0.09-3.23) |  |  | 2 (0.5) | 0.26 (0.04-1.53) |  | 0.24 (0.04-1.43) |  |
| ≥1 time/week | 459 (45.7) | 6 (1.3) | 1.00 (0.20-4.94) |  | 1.10 (0.21-5.70) |  |  | 6 (1.3) | 0.64 (0.16-2.56) |  | 0.72 (0.17-2.98) |  |
| **Salted fish** |  |  |  |  |  |  |  |  |  |  |  |  |
| Almost never | 311 (27.0) | 5 (1.6) | Ref. | 0.9086 | Ref. | 0.7287 |  | 5 (1.6) | Ref. | 0.6126 | Ref. | 0.8481 |
| ≤2–3 times/month | 431 (37.4) | 3 (0.7) | 0.40 (0.10-1.66) |  | 0.43 (0.10-1.80) |  |  | 2 (0.5) | 0.27 (0.05-1.37) |  | 0.30 (0.06-1.56) |  |
| ≥1 time/week | 411 (35.7) | 7 (1.7) | 0.98 (0.31-3.07) |  | 1.22 (0.37-4.03) |  |  | 5 (1.2) | 0.69 (0.20-2.38) |  | 0.97 (0.27-3.47) |  |
| **Red meat** |  |  |  |  |  |  |  |  |  |  |  |  |
| Almost never | 181 (24.2) | 3 (1.7) | Ref. | 0.8705 | Ref. | 0.8275 |  | 2 (1.1) | Ref. | 0.3929 | Ref. | 0.5591 |
| ≤2–3 times/month | 345 (46.1) | 6 (1.7) | 0.90 (0.23-3.61) |  | 1.17 (0.28-4.83) |  |  | 4 (1.2) | 0.95 (0.17-5.16) |  | 1.20 (0.21-6.79) |  |
| ≥1 time/week | 223 (29.8) | 4 (1.8) | 0.88 (0.20-3.94) |  | 1.22 (0.26-5.68) |  |  | 1 (0.5) | 0.35 (0.03-3.87) |  | 0.50 (0.04-5.86) |  |
| **Beef** |  |  |  |  |  |  |  |  |  |  |  |  |
| Almost never | 396 (52.9) | 7 (1.8) | Ref. | 0.9964 | Ref. | 0.9057 |  | 4 (1.0) | Ref. | 0.9130 | Ref. | 0.9965 |
| ≤2–3 times/month | 268 (35.8) | 4 (1.5) | 0.77 (0.22-2.62) |  | 0.86 (0.24-3.07) |  |  | 2 (0.8) | 0.71 (0.13-3.87) |  | 0.73 (0.12-4.26) |  |
| ≥1 time/week | 85 (11.4) | 2 (2.4) | 1.20 (0.25-5.77) |  | 1.17 (0.24-5.77) |  |  | 1 (1.2) | 1.10 (0.12-9.87) |  | 1.09 (0.11-10.91) |  |
| **Pork** |  |  |  |  |  |  |  |  |  |  |  |  |
| Almost never | 210 (28.1) | 3 (1.4) | Ref. | 0.8228 | Ref. | 0.8074 |  | 2 (1.0) | Ref. | 0.5317 | Ref. | 0.7581 |
| ≤2–3 times/month | 331 (44.3) | 7 (2.1) | 1.32 (0.34-5.10) |  | 1.73 (0.44-6.79) |  |  | 4 (1.2) | 1.16 (0.21-6.33) |  | 1.57 (0.28-8.77) |  |
| ≥1 time/week | 207 (27.7) | 3 (1.5) | 0.85 (0.17-4.20) |  | 1.24 (0.24-6.36) |  |  | 1 (0.5) | 0.44 (0.04-4.88) |  | 0.71 (0.06-8.32) |  |
| **Soybean/tofu** |  |  |  |  |  |  |  |  |  |  |  |  |
| Almost never | 41 (5.0) | 1 (2.4) | Ref. | 0.7896 | Ref. | 0.8517 |  | 1 (2.4) | Ref. | 0.4225 | Ref. | 0.3560 |
| ≤2–3 times/month | 276 (33.4) | 3 (1.1) | 0.39 (0.04-3.73) |  | 0.55 (0.06-5.37) |  |  | 4 (1.5) | 0.55 (0.06-4.91) |  | 0.66 (0.07-6.14) |  |
| ≥1 time/week | 509 (61.6) | 8 (1.6) | 0.65 (0.08-5.19) |  | 0.67 (0.08-5.39) |  |  | 5 (1.0) | 0.41 (0.05-3.47) |  | 0.48 (0.05-4.35) |  |
| 1) Crude | | | | | | | | | | | | |
| 2) Adjusted for age, education, marital status, BMI | | | | | | | | | | | | |
